# Associations of reproductive factors with breast cancer prognosis and the modifying effects of menopausal status

**DOI:** 10.1002/cam4.2707

**Published:** 2019-11-14

**Authors:** Jia‐Yi Zhang, Mei‐Xia Wang, Xiang Wang, Yue‐Lin Li, Zhuo‐Zhi Liang, Ying Lin, Qiang Liu, Xiao‐Ming Xie, Lu‐Ying Tang, Ze‐Fang Ren

**Affiliations:** ^1^ School of Public Health Sun Yat‐sen University Guangzhou China; ^2^ Xiamen Branch Zhongshan Hospital Fudan University Xiamen China; ^3^ The First Affiliated Hospital Sun Yat‐sen University Guangzhou China; ^4^ The Second Affiliated Hospital Sun Yat‐sen University Guangzhou China; ^5^ The Sun Yat‐sen Cancer Center Guangzhou China; ^6^ The Third Affiliated Hospital Sun Yat‐sen University Guangzhou China

**Keywords:** breast cancer, menopausal status, prognosis, reproductive factors

## Abstract

Reproductive factors associated with breast cancer risk may also affect the prognosis. This study aimed to evaluate the associations of multiple reproductive factors with breast cancer prognosis and the modifying effects of menopausal status. We obtained data from 3805 breast cancer patients recruited between October 2008 and June 2016 in Guangzhou. The subjects were followed up until 30 June 2018. The hazard ratios (HRs) and 95% confidence intervals (95% CIs) were calculated using multivariate Cox models to estimate the associations. It was found that there were U‐shaped patterns for the associations of age at first birth and durations from first/last birth to diagnosis with breast cancer prognosis. The adverse effects of old age at first birth [>30 years vs 23‐30 years, HR (95% CI): 1.59 (1.01‐2.50)] and long intervals from first [≥20 years vs 10‐19 years, HR (95% CI): 1.55 (1.07‐2.27)] or last [≥20 years vs 10‐19 years, HR (95% CI): 1.63 (1.08‐2.46)] birth to diagnosis on progression‐free survival (PFS) were significantly more pronounced among premenopausal women. Additionally, long interval (>5 years) between first and second birth was associated with a better PFS [HR (95% CI): 0.64 (0.42‐0.97)]. These results suggested that age at first birth, durations from first/last birth to diagnosis, and intervals between first and second birth should be taken into account when following the patients and assessing the prognosis of breast cancer, particularly for premenopausal patients. These findings would also have implications for further insight into the mechanisms of breast cancer development.

## Graphical abstract

This study found U‐shaped patterns for the associations of age at first birth and durations from first/last birth to diagnosis with breast cancer prognosis, particularly for premenopausal women; a relatively long interval between first and second birth may benefit prognosis of breast cancer.

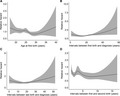

## 1 INTRODUCTION

Breast cancer is the most common cancer and one of the leading causes of cancer death among females worldwide.^1^ Reproductive factors, such as age at first birth, time intervals from a birth to diagnosis, and number of parity, were believed to be closely associated with the initiation of breast cancer.^2^, ^3^, ^4^ The potential biological mechanisms underlying the associations included increased circulating hormones, substantial expansion of the epithelial trees, extensive stromal remodeling, and pro‐inflammatory and wound‐healing changes in the microenvironment.^5^, ^6^ These mechanisms were reported to influence the progression of breast cancer.^7^, ^8^, ^9^ Therefore, it is possible that the reproductive factors may also affect the prognosis. A few previous studies have explored the associations, but the results were inconsistent.^10^, ^11^ For example, some have reported that early age at first birth^12^, ^13^, ^14^ and recent birth^15^, ^16^, ^17^, ^18^, ^19^ were at an increased risk of progression, whereas others have shown that late age at first birth^15^, ^20^, ^21^ and long intervals from last birth to diagnosis^22^ were related to a poor prognosis. Moreover, most of these studies were conducted in Western countries; few of them were in Asia^14^, ^17^, ^18^, ^21^, ^22^, ^23^; no study was performed in China. Meanwhile, the distributions of reproductive factors and breast cancer incidence among women in China were quite different from those in Western countries, such as younger age at first birth, the unique one‐child policy, and more premenopausal than postmenopausal patients of breast cancer,^24^, ^25^ which may have different effects on the prognosis.

In addition, two previous studies mentioned that the associations between reproductive factors and breast cancer survival might be nonlinear relationship,^15^, ^23^ but no study has assessed this relationship. Furthermore, menopausal status was related to the levels of hormones,^26^ which may interact with the hormonal changes associated with pregnancy^27^, ^28^ to affect breast cancer prognosis. However, most of the previous studies mixed patients with different menopausal status, and the interactions with reproductive factors have not been explored.

Therefore, this study was aimed to explore the associations of reproductive factors with breast cancer prognosis, applying restricted cubic spline to explore the nonlinear relationship, and further investigate whether the associations were modified by menopausal status using the data from the Guangzhou Breast Cancer Study (GZBCS).

## 2 MATERIALS AND METHODS

### 2.1 Study population

A total of 4114 subjects were recruited between October 2008 and June 2016 from the First and the Second Affiliated Hospitals and the Cancer Center of Sun Yat‐sen University in Guangzhou, China. The details were described elsewhere.^29^ Patients who were pathologically confirmed primary breast cancer and reported reproductive history were eligible for this study (N = 3935). Patients with stage IV or with a history of other cancers were excluded, yielding an analytic sample of 3805 cases. The informed consents were obtained from each participant. This study was approved by the Ethical Committee of the School of Public Health at Sun Yat‐sen University.

### 2.2 Data collection

When first hospitalized for primary breast cancer, the subjects were interviewed in‐person by trained investigators using a structured questionnaire. We collected the following information: demographic characteristics, menstrual and reproductive history, life styles (physical exercise, tea consumption, smoking etc), and family history of breast cancer. Reproductive history included total number of pregnancies, the outcome of each pregnancy, and age at each birth.

Clinicopathological characteristics and body mass index (BMI) were collected from medical records. The status of estrogen receptor (ER), progesterone receptor (PR), and human epidermal growth factor receptor 2 (HER2) was determined by pathologists using immunohistochemical tests. Detailed definitions of ER, PR, and HER2 status were previously described in detail.^30^

### 2.3 Follow‐up

All participants were followed up at least every 3 months during the first year, and every 6 months during the second and the third year; thereafter, patients were followed up once every year until death or 30 June 2018. The follow‐up data, including survival condition (death, recurrence, metastasis, or other newly diagnosed diseases), updated contact information, postdiagnostic life style, and treatment information, were obtained from 3273 (86.1%) breast cancer patients by means of phone call and outpatient department visits. The endpoints of this study were overall survival (OS) and progression‐free survival (PFS), defined as the time from diagnosis until death and the time from diagnosis to the date of progression (including recurrence, metastasis, or death), respectively. Survival status was censored at the date of the latest interview or 30 June 2018.

### 2.4 Statistical analysis

Log‐rank test was used to estimate the associations of breast cancer prognosis with demographic and clinical characteristics at baseline to determine the potential confounders. To explore the nonlinear effect on breast cancer survival, the maternal age‐related factors (age at first birth, intervals from first/last birth to diagnosis, and intervals between first and second birth) were modeled as continuous variables and fitted in a Cox proportional hazard model using restricted cubic splines with knots at the 5th, 35th, 65th, and 95th percentiles. The classifications of those variables were then defined based on the results of the restricted cubic spline for breast cancer PFS. Cox proportional hazard models were performed to investigate the associations between reproductive characteristics and breast cancer prognosis with hazard ratios (HRs) and 95% confidence intervals (95% CIs), adjusting for age at diagnosis, clinical stage, education, menopausal status, ER, and HER2 status.

To assess the joint effects of different reproductive factors on breast cancer prognosis, we cross‐classified the patients by different reproductive factors. Further stratification analyses were performed by menopausal status (premenopausal vs postmenopausal) and ER status (positive vs negative) to see whether the associations were modified by clinicopathological characteristics. Interactions between those factors were estimated by the product terms in the Cox regression models. All analyses were performed with R software version 3.4 with a two‐sided significance level of *P* < .05.

### 2.5 Data availability

The data that support the findings of this study are available on request from the corresponding author.

## 3 RESULTS

### 3.1 Demographic and clinicopathological characteristics and the associations with breast cancer prognosis

As shown in Table S1, the median age at diagnosis was 47 years (interquartile range: 40‐55 years). Nearly half of the subjects were underneath junior school (49.5%) and more than half of them were premenopausal (62.8%). Most women were ER‐positive (75.3%) and almost four‐fifths of them were diagnosed at early stage (stage I/II: 79.7%). Over 50 months (median) follow‐up, a total of 236 patients died and 442 patients experienced breast cancer progression. Log‐rank test showed that educational level, clinical stage, menopausal status, ER, and HER2 status were significantly associated with breast cancer prognosis.

### 3.2 Associations of reproductive factors with breast cancer prognosis and the modifying effects of menopausal status

**Figure 1:**
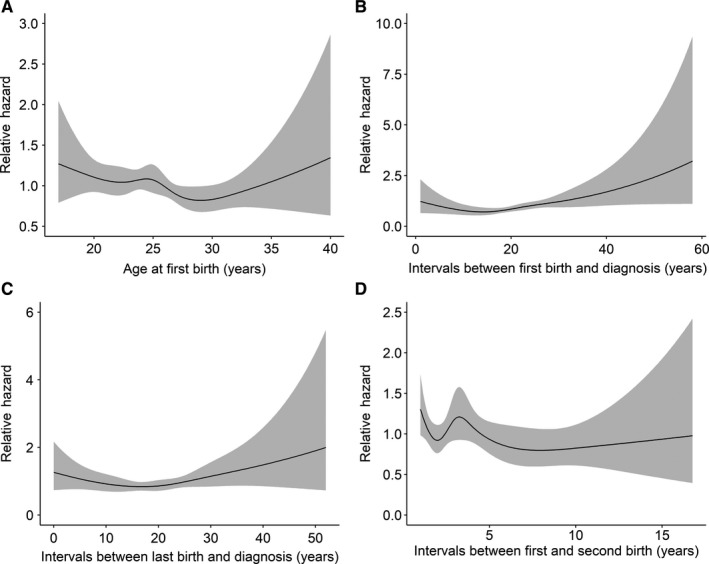
The restricted cubic splines of maternal age‐related factors with breast cancer progression‐free survival, hazard ratio adjusted for age at diagnosis; Gray represents the 95% confidence interval: (A) age at first birth, (B) intervals between first birth and diagnosis, (C) intervals between last birth and diagnosis, and (D) intervals between first and second birth

**Figure 2:**
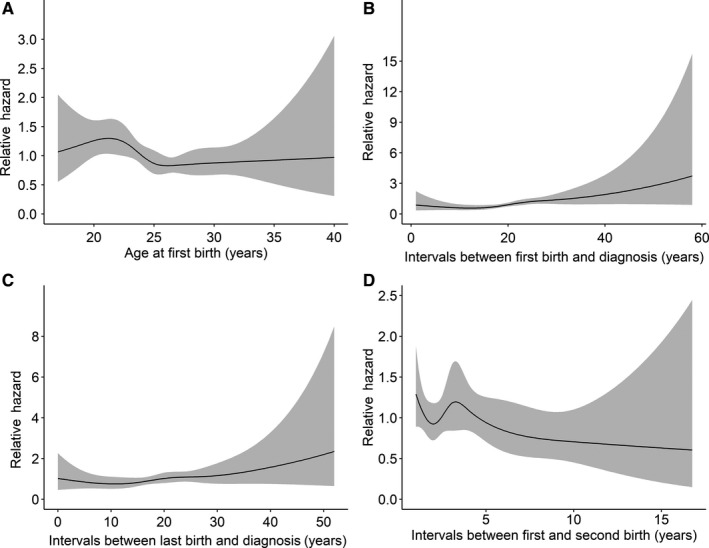
The restricted cubic splines of maternal age‐related factors with breast cancer overall survival, hazard ratio adjusted for age at diagnosis; Gray represents the 95% confidence interval: (A) age at first birth, (B) intervals between first birth and diagnosis, (C) intervals between last birth and diagnosis, and (D) intervals between first and second birth

Restricted cubic spline analyses were used to estimate the nonlinear relationship of maternal age‐related factors with breast cancer prognosis (Figures 1, 2). For PFS, an obvious U‐shape relation was shown for age at first birth, though the *P*‐value was not statistically significant (Figure 1A, *P*_nonlinear_ = .382); there was also a pronounced U‐shape association for the intervals between first/last birth and diagnosis (Figure 1B,C *P*_nonlinear_ = .005 and .028, respectively). For OS, the patterns were similar to that of PFS (Figure 2A‐C). The nonlinear correlation of the intervals between first and second birth with the prognosis was not significant (Figures 1D, 2D, *P*_nonlinear_ = .215 and .540, respectively).

We then categorized the variables according to the nonlinear relationship patterns and fitted them in the Cox proportional hazard models to examine the associations with breast cancer prognosis (Tables 1, 2). For age at first birth, compared to the women at 23‐30 years old, those at older than 30 years had a nonsignificant slight increase in progression [HR (95% CI): 1.20 (0.82, 1.75)]; the association turned to be significant among premenopausal women [HR (95% CI): 1.59 (1.01‐2.50)] but not postmenopausal women [HR (95% CI): 0.75 (0.36‐1.55)], and the interaction was significant (*P*_interaction_ = .048). Similarly, for the intervals between first/last birth and diagnosis, the longer intervals (≥20 years) were nonsignificantly associated with a poor prognosis (OS and PFS) as compared to the intervals of 10 to 19 years (Table 1), while the associations with PFS were significant among premenopausal women [HR (95% CI): 1.55 (1.07‐2.27) for first birth to diagnosis and 1.63 (1.08‐2.46) for last birth to diagnosis], and the interactions were significant (*P*_interaction_ = .048 and .040, respectively, Table 2). As for intervals between first and second birth, patients who had long intervals (>5 years) experienced a significantly reduced risk of progression [HR (95% CI): 0.64 (0.42‐0.97), Table 1], while there was no differential association across menopausal status (Table 2). For parity, abortion, and the numbers, no significant association with the prognosis was observed.

**Table 1:** Associations between reproductive factors and breast cancer prognosis

Variables	Overall survival			Progression‐free survival		
	Fatality (%)	HR (95% CI)^a^	HR (95% CI)^b^	Progression (%)	HR (95% CI)^a^	HR (95% CI)^b^
Age at first birth (y)						
<23	63 (9.7)	**1.46 (1.08, 1.97)**	1.29 (0.93, 1.80)	102 (16.1)	1.17 (0.93, 1.47)	1.11 (0.87, 1.43)
23~30	134 (7.2)	1.00 (reference)	1.00 (reference)	270 (14.8)	1.00 (reference)	1.00 (reference)
>30	20 (8.3)	1.28 (0.80, 2.05)	1.44 (0.87, 2.38)	37 (15.2)	1.15 (0.81, 1.62)	1.20 (0.82, 1.75)
Intervals between first birth and diagnosis (y)						
<10	22 (6.9)	1.36 (0.76, 2.46)	1.63 (0.87, 3.05)	58 (18.4)	1.43 (0.98, 2.07)	1.49 (1.00, 2.22)
10~19	46 (5.6)	1.00 (reference)	1.00 (reference)	108 (13.4)	1.00 (reference)	1.00 (reference)
≥20	149 (9.2)	**1.71 (1.14, 2.55)**	1.50 (0.94, 2.41)	242 (15.4)	1.22 (0.92, 1.60)	1.25 (0.90, 1.74)
Intervals between last birth and diagnosis (y)						
<10	34 (6.7)	1.36 (0.78, 2.37)	1.24 (0.69, 2.24)	88 (17.5)	1.40 (0.99, 2.00)	1.27 (0.87, 1.85)
10~19	34 (5.4)	1.00 (reference)	1.00 (reference)	80 (13.0)	1.00 (reference)	1.00 (reference)
≥20	127 (9.2)	**1.84 (1.19, 2.84** **)**	1.60 (0.95, 2.68)	204 (15.2)	1.25 (0.93, 1.68)	1.26 (0.88, 1.80)
Intervals between first and second birth (y)						
≤5	121 (10.2)	1.00 (reference)	1.00 (reference)	204 (17.6)	1.00 (reference)	1.00 (reference)
>5	18 ( 6.3)	0.60 (0.36, 1.00)	0.71 (0.41, 1.22)	36 (12.6)	0.69 (0.48, 1.00)	**0.64 (0.42, 0.97)**
Parity						
0	16 (9.1)	1.20 (0.72, 2.03)	1.01 (0.54, 1.88)	25 (14.6)	0.93 (0.61, 1.40)	0.94 (0.60, 1.47)
1~2	162 (7.3)	1.00 (reference)	1.00 (reference)	309 (14.2)	1.00 (reference)	1.00 (reference)
≥3	58 (9.9)	**1.38 (1.01, 1.88)**	0.96 (0.68, 1.36)	108 (19.0)	**1.42 (1.13, 1.78)**	1.07 (0.83, 1.40)
Abortion						
Never	102 (8.0)	1.00 (reference)	1.00 (reference)	192 (15.5)	1.00 (reference)	1.00 (reference)
Ever	125 (8.0)	0.90 (0.69, 1.17)	0.98 (0.74, 1.31)	228 (14.9)	0.91 (0.75, 1.11)	0.92 (0.75, 1.13)
No. of abortions						
0	102 (8.0)	1.00 (reference)	1.00 (reference)	192 (15.5)	1.00 (reference)	1.00 (reference)
1	64 (8.2)	0.92 (0.67, 1.26)	1.02 (0.73, 1.43)	109 (14.3)	0.86 (0.68, 1.10)	0.90 (0.70, 1.15)
≥2	61 (7.8)	0.89 (0.65, 1.22)	0.95 (0.67, 1.34)	119 (15.4)	0.96 (0.76, 1.21)	0.95 (0.74, 1.22)

Abbreviations: ER, estrogen receptor; HER2, human epidermal growth factor receptor 2; HR, hazard ratio.

Bold character indicates statistically significant result.

a Adjusted for age at diagnosis.

b Adjusted for age at diagnosis, education, menopausal status, clinical stage, ER status, HER2 status.

**Table 2:** Associations between reproductive factors and breast cancer PFS stratified by menopausal status

Variables	Premenopause		Postmenopause		*P*_interaction_
	Progression (%)	HR (95% CI)^a^	Progression (%)	HR (95% CI)^a^	
Age at first birth (y)					
<23	52 (14.1)	1.14 (0.81, 1.60)	50 (20.0)	1.12 (0.78, 1.63)	0.870
23~30	149 (13.4)	1.00 (reference)	109 (16.3)	1.00 (reference)	
>30	26 (16.8)	**1.59 (1.01, 2.50)**	9 (11.4)	0.75 (0.36, 1.55)	**0.048**
Intervals between first birth and diagnosis (y)					
<10	56 (18.7)	**1.53 (1.02, 2.30)**	0 (0.0)	—	—
10~19	87 (12.1)	1.00 (reference)	13 (21.3)	1.00 (reference)	
≥20	84 (13.6)	**1.55 (1.07, 2.27)**	155 (16.6)	0.70 (0.37, 1.31)	**0.048**
Intervals between last birth and diagnosis (y)					
<10	83 (17.4)	1.38 (0.93, 2.05)	1.0 (25.0)	—	—
10~19	62 (11.4)	1.00 (reference)	12 (21.4)	1.00 (reference)	
≥20	59 (13.3)	**1.63 (1.08, 2.46)**	142 (16.2)	0.70 (0.37, 1.33)	**0.040**
Intervals between first and second birth (y)					
≤5	94 (16.5)	1.00 (reference)	106 (18.7)	1.00 (reference)	
>5	24 (11.5)	0.70 (0.43, 1.15)	9 (13.4)	0.54 (0.23, 1.23)	0.565
Parity					
0	22 (16.3)	0.91 (0.56, 1.49)	3 (12.5)	0.91 (0.28, 2.90)	0.854
1~2	192 (13.5)	1.00 (reference)	106 (15.2)	1.00 (reference)	
≥3	39 (16.5)	0.98 (0.66, 1.46)	66 (20.6)	1.06 (0.74, 1.52)	0.500
Abortion					
Never	105 (13.5)	1.00 (reference)	82 (19.3)	1.00 (reference)	
Ever	137 (14.3)	1.08 (0.82, 1.41)	82 (15.0)	0.78 (0.56, 1.09)	0.087

Abbreviations: ER, estrogen receptor; HER2, human epidermal growth factor receptor 2; HR, hazard ratio; PFS, progression‐free survival.

a Adjusted for age at diagnosis, education, clinical stage, ER status, HER2 status; Bold character indicates statistically significant result.

### 3.3 Joint effects of reproductive factors on breast cancer PFS

We further examined the joint effects of multiple reproductive factors (Table 3). Long intervals between first and second birth were related to a reduced risk of disease progression among patients with late age at first birth [HR (95% CI): 0.52 (0.31‐0.89)], whereas the association was not significant among patients with first birth before 23 years [HR (95% CI): 0.89 (0.46‐1.75)], but the interaction was not significant (*P*_interaction_ = .351). There were no marked joint effects across other reproductive factors on the PFS either.

**Table 3:** Joint effects of reproductive factors on breast cancer PFS

Variables			Progression (%)	HR (95% CI)^a^
Age at first birth (y)	Intervals between first and second birth (y)			
<23	≤5	71 (16.8)		1.00 (reference)
	>5	11 (12.8)		0.89 (0.46, 1.75)
≥23	≤5	133 (18.1)		1.00 (reference)
	>5	25 (12.6)		**0.52 (0.31, 0.89)**
P for interaction	0.351			
Age at first birth (y)	Intervals between first birth and diagnosis (y)			
<23	<10	5 (26.3)		3.07 (1.00, 9.44)
	10~19	20 (16.7)		1.00 (reference)
	≥20	77 (15.7)		1.33 (0.39, 4.55)
≥23	<10	54 (18.1)		1.54 (0.96, 2.45)
	10~19	88 (12.9)		1.00 (reference)
	≥20	165 (15.3)		1.29 (0.89, 1.86)
P for interaction	0.205^b^/0.846^c^			
Age at first birth (y)	Intervals between last birth and diagnosis (y)			
<23	<10	10 (16.4)		1.21 (0.50, 2.94)
	10~19	15 (18.5)		1.00 (reference)
	≥20	65 (16.1)		1.90 (0.47, 7.66)
≥23	<10	78 (17.7)		1.40 (0.89, 2.21)
	10~19	65 (12.2)		1.00 (reference)
	≥20	138 (14.7)		1.28 (0.86, 1.92)
P for interaction	0.616^b^/0.599^c^			

Abbreviations: HR, hazard ratio; PFS, progression‐free survival.

a Adjusted for age at diagnosis, education, menopausal status, clinical stage, ER status, HER2 status.

b Between age at first birth and intervals between birth and diagnosis (<10 y vs 10~19 y).

c Between age at first birth and intervals between birth and diagnosis (>20 y vs 10~19 y); Bold character indicates statistically significant result.

## 4 DISCUSSION

In this study, we found that there were U‐shaped patterns for the effects of age at first birth and age intervals from first/last birth to diagnosis on the prognosis of breast cancer; particularly, the adverse effects of late age at first birth and long intervals from first/last birth to diagnosis were more pronounced among premenopausal women than postmenopausal women, and the interactions were significant. Additionally, long interval between first and second birth was related to a better prognosis of breast cancer.

The results from studies on age at first birth as a prognostic factor have been debatable. Some have reported no association,^11^, ^16^ and others have reported a poorer survival with either early age^12^, ^13^, ^14^ or late age at first birth.^15^, ^20^, ^21^ In the present study, a nonlinear relationship between breast cancer prognosis and age at first birth was observed, which may to some extent explain the results from those previous studies. It was reported that early birth was associated with a worse socioeconomic status,^2^, ^31^, ^32^ which could be a reason for the association between early birth and poorer prognosis. In addition, the levels of estrogens and androgens were higher in younger than older pregnant women,^33^, ^34^ while the elevated hormones have been shown to increase cell division and stimulate the growth of breast cancer cells,^35^, ^36^ resulting in a more aggressive biology of breast cancer. As for the worse prognosis of late age, one of the reasons might be that women with birth at a late age had an increased expression of cyclin D1 and reduced expressions of p27 and E‐cadherin protein^37^, ^38^ that were related to an aggressive tumor behavior.^39^, ^40^, ^41^

The results from previous studies on breast cancer prognosis associated with the intervals between first/last birth and diagnosis were not consistent either. While several studies have reported that women who had given birth within 2‐5 years before diagnosis of breast cancer were in relation to an increased mortality,^15^, ^17^, ^19^, ^42^ other studies have shown that a diagnosis up to 10 years after birth was associated with decreased survival.^12^, ^16^ Meanwhile, a study conducted in Malaysia demonstrated an increase in risk of mortality with an even longer interval (>36 years).^22^ It should be noticed that these previous studies arbitrarily applied various cutoff values of the intervals, which might contribute to the discrepancies. We applied restricted cubic splines to accordingly define the cutoff values and found that women diagnosed within 10 years or more than 20 years after a birth had a poor prognosis compared with the group of 10‐19 years. Asztalos et al showed an increase in inflammatory activity in the post‐pregnancy breast as suggested by the upregulation of numerous inflammation‐related genes (LBP, SAA1/2, and CCL21) and this response could last for 10 years,^43^ which might explain the adverse effect of the diagnosis within 10 years after a birth. The increased progression among the longer interval group (≥20 years) might be explained by their low immunity and comorbidities due to old age.

Interestingly, we further found that the above effects of late first birth and greater intervals from first/last birth to diagnosis were more evident among premenopausal patients. The underlying biologic mechanisms were not clear and possibly involved the different hormonal milieu in premenopausal patients who had high concentrations of circulating estrogens and progesterone.^44^, ^45^, ^46^ It was reported that women with later age at first birth would take a longer time to recover because they had greater immunosuppression or stronger inflammatory response.^47^ Meanwhile, estrogens could enhance immunosuppression through inhibition of natural killer and cytotoxic T lymphocyte‐mediated tumor cell elimination.^48^ Therefore, late first birth might interact with menopausal status to suppress the immune function, leading to an increased chance for the tumor cells to proliferate and metastasize. Further explorations on the exact mechanisms are needed.

It is worth noting that a long interval between first and second birth was independently associated with a reduced risk of progression. Previous studies on breast cancer risk have shown that a short interval between first and second birth was associated with a significantly increased risk of breast cancer,^49^, ^50^ which supported our finding to some extent. A possible mechanism was that the mammary cells were repeatedly exposed to high amounts of estrogens and other steroids in closely occurring births, which may stimulate growth and promotion of occult tumor cells.^36^ Moreover, this effect was expected to specifically influence ER‐positive tumors,^6^ in line with our result (Table S2) that a long interval between first and second birth was associated with a stronger effect on ER+ breast cancer [HR (95% CI): 0.41 (0.22‐0.75)] than ER‐ counterparts [HR (95% CI): 1.02 (0.55, 1.88)].

The present study examined the separate and joint effects of multiple reproductive characteristics on breast cancer prognosis in China, which added valuable evidence to improve our understanding on the prognostic impact of reproductive factors on breast cancer. With further stratification analyses, we were able to detect the effect modification by ER and menopausal status. Nevertheless, only PFS was applied in the stratification analyses as the number of deaths was limited, though the progression was able to reflect the survival condition of patients. In addition, the numbers of subjects with short intervals from first/last birth to diagnosis were relatively small and the corresponding interactions were not able to be evaluated. Thus, the findings should be interpreted cautiously. Finally, there was a lack of information on prior use of hormonal therapy and socioeconomic status in our study, which may have confounded the results; however, the adjustment for education and clinicopathological characteristics partly compensated this defect.

In conclusion, it was found that the effects of age at first birth and durations from first/last birth to diagnosis on breast cancer prognosis occurred in U‐shaped patterns instead of linear relationships as suggested in previous studies, which should be taken into account when following the patients and assessing the prognosis of breast cancer, particularly for premenopausal patients. These findings would also have implications for further insight into the mechanisms of breast cancer development.

## CONFLICT OF INTEREST

None declared.
