# Supplementary material for: Associations of reproductive factors with breast cancer prognosis and the modifying effects of menopausal status
Source: Cancer Med. 2019 Nov 14;9(1):385–93. doi: 10.1002/cam4.2707 (PMC6943140; doi:10.1002/cam4.2707)
Supplement: Supplementary file 1 [file CAM4-9-385-s001.doc]

| **Supplementary Table 1** Demographic and clinicopathological characteristics and the associations with breast cancer prognosis | | | | | | |
| --- | --- | --- | --- | --- | --- | --- |
| Characteristics | Total (%) | Overall Survival | |  | Progression-free Survival | |
|  |  | Fatality (%) | *P* value ^a^ |  | Progress (%) | *P* value ^a^ |
| Age at diagnosis |  |  |  |  |  |  |
| <40 | 766 (23.1) | 52 (7.5) |  |  | 118 (17.3) |  |
| 40~59 | 2084 (62.7) | 146 (7.8) | 0.500 |  | 260 (14.2) | 0.300 |
| ≥ 60 | 473 (14.2) | 38 (9.2) |  |  | 64 (16.1) |  |
| Education |  |  |  |  |  |  |
| Junior and below | 1575 (49.5) | 137 (9.7) |  |  | 232 (16.9) |  |
| Senior | 803 (25.2) | 49 (6.7) | **0.001** |  | 96 (13.5) | **0.010** |
| College and above | 807 (25.3) | 43 (6.0) |  |  | 94 (13.3) |  |
| BMI (kg/m2) |  |  |  |  |  |  |
| <18.5 | 187 ( 5.8) | 13 (7.9) |  |  | 29 (18.5) |  |
| 18.5-23.9 | 1871 (58.0) | 132 (7.9) | 0.800 |  | 345 (14.9) | 0.600 |
| ≥24 | 1169 (36.2) | 87 (8.3) |  |  | 156 (15.4) |  |
| Menopause |  |  |  |  |  |  |
| Pre | 2025 (62.8) | 124 (6.8) | **0.007** |  | 253 (14.1) | 0.060 |
| Post | 1200 (37.2) | 105 (9.8) |  |  | 175 (16.8) |  |
| ER |  |  |  |  |  |  |
| Negative | 777 (24.7) | 92 (13.0) | **< 0.001** |  | 148 (21.4) | **< 0.001** |
| Positive | 2364 (75.3) | 130 (6.1) |  |  | 270 (13.0) |  |
| HER2 |  |  |  |  |  |  |
| Negative | 2447 (80.0) | 163 (7.4) | **0.004** |  | 316 (14.6) | **0.010** |
| Positive/Equivocal | 612 (20.0) | 53 (9.7) |  |  | 92 (17.3) |  |
| Clinical stage |  |  |  |  |  |  |
| I/II | 2654 (79.7) | 138 (5.8) | **< 0.001** |  | 268 (11.5) | **< 0.001** |
| III | 674 (20.3) | 98 (16.2) |  |  | 174 (29.8) |  |
| ^a^ Log-rank test; Bold character indicate statistically significant result. | | | | | | |

| **Supplementary Table 2** Associations between reproductive factors and breast cancer PFS stratified by ER status | | | | | | |
| --- | --- | --- | --- | --- | --- | --- |
| Variables | ER- | |  | ER+ | | ***P* _interaction_** |
|  | Progression (%) | HR (95% CI) ^a^ |  | Progression (%) | HR (95% CI) ^a^ |  |
| Age at first birth (years) | | |  |  |  |  |
| <23 | 31 (18.3) | 0.78 (0.51, 1.20) |  | 66 (15.3) | 1.37 (1.00, 1.87) | 0.099 |
| 23~30 | 96 (22.6) | 1.00 (reference) |  | 160 (12.2) | 1.00 (reference) |  |
| >30 | 9 (18.0) | 1.24 (0.62, 2.50) |  | 26 (14.8) | 1.21 (0.77, 1.91) | 0.887 |
| Intervals between first birth and diagnosis (years) | | |  |  |  |  |
| <10 | 16 (19.8) | 1.70 (0.81, 3.57) |  | 38 (18.2) | 1.40 (0.86, 2.27) | 0.839 |
| 10~19 | 32 (19.4) | 1.00 (reference) |  | 71 (11.9) | 1.00 (reference) |  |
| ≥20 | 88 (22.1) | 0.95 (0.52, 1.74) |  | 143 (12.9) | 1.44 (0.97, 2.15) | 0.683 |
| Intervals between last birth and diagnosis (years) | | |  |  |  |  |
| <10 | 27 (20.6) | 1.36 (0.68, 2.72) |  | 56 (16.5) | 1.22 (0.77, 1.92) | 0.667 |
| 10~19 | 23 (19.5) | 1.00 (reference) |  | 53 (11.4) | 1.00 (reference) |  |
| ≥20 | 75 (22.1) | 0.87 (0.46, 1.64) |  | 122 (12.9) | 1.52 (0.98, 2.37) | 0.888 |
| Intervals between first and second birth (years) | | |  |  |  |  |
| ≤5 | 73 (24.3) | 1.00 (reference) |  | 118 (14.8) | 1.00 (reference) |  |
| >5 | 17 (23.0) | 1.02 (0.55, 1.88) |  | 17 (8.5) | **0.41 (0.22, 0.75)** | 0.069 |
| Parity |  |  |  |  |  |  |
| 0 | 9 (25.0) | 1.22 (0.58, 2.58) |  | 14 (11.4) | 0.86 (0.49, 1.52) | 0.902 |
| 1~2 | 98 (19.4) | 1.00 (reference) |  | 199 (12.7) | 1.00 (reference) |  |
| ≥3 | 41 (27.3) | 0.96 (0.62, 1.48) |  | 57 (15.0) | 1.12 (0.81, 1.57) | 0.955 |
| Abortion |  |  |  |  |  |  |
| Never | 64 (21.9) | 1.00 (reference) |  | 117 (13.4) | 1.00 (reference) |  |
| Ever | 75 (20.9) | 0.91 (0.64, 1.30) |  | 144 (13.0) | 0.92 (0.71, 1.19) | 0.877 |
| ^a^ Adjusted for age at diagnosis, education, menopausal status, clinical stage, HER2 status; Bold character indicate statistically significant result. | | | | | | |
